# COVID-19, disability and the context of healthcare triage in South Africa: Notes in a time of pandemic

**DOI:** 10.4102/ajod.v9i0.766

**Published:** 2020-08-18

**Authors:** Emma L. McKinney, Victor McKinney, Leslie Swartz

**Affiliations:** 1 Interdisciplinary Centre for Sports Science and Development, Community and Health Sciences, University of the Western Cape, Cape Town, South Africa; 2 Department of Health and Rehabilitation Sciences, Faculty of Health Sciences, University of Cape Town, Cape Town, South Africa; 3 Department of Psychology, Faculty of Arts and Social Sciences, Stellenbosch University, Cape Town, South Africa

**Keywords:** COVID-19, disabled people, triage policies, ventilators, ICU admission, ethics of care, accessibility, South Africa

## Abstract

During disasters, when resources and care are scarce, healthcare workers are required to make decisions and prioritise which patients receive life-saving resources over others. To assist healthcare workers in standardising resources and care, triage policies have been developed. However, the current COVID-19 triage policies and practices in South Africa may exclude or disadvantage many disabled people, especially people with physical and intellectual impairments, from gaining intensive care unit (ICU) access and receiving ventilators if becoming ill. The exclusion of disabled people goes against the principles established in South Africa’s Constitution, in which all people are regarded as equal, have the right to life and inherent dignity, the right to access healthcare, as well as the protection of dignity. In addition, the triage policy contravenes the United Nations Convention on the Rights of Persons with Disabilities, which the South African government has signed and ratified. This article raises debates about whose lives matter and whose lives are ‘worth’ saving over others, and although the focus is on South Africa, the issues may be relevant to other countries where life-saving resources are being rationed.

## Background

Disabled people experience discrimination and hardship in all spheres of life, including employment, education and access to healthcare. In addition, disabled people are more likely to experience increased health needs, worse health outcomes and discriminatory laws, as well as stigma. These issues are likely to be intensified during the COVID-19 epidemic (Armitage & Nellums 2020; Kittay 2020; Kuper et al. 2020). According to the United Nations (UN), disabled people are ‘disproportionately impacted by the COVID-19 outbreak’ (UN 2020:4). During disasters and epidemics, demand for life-saving medical equipment and interventions increases significantly, and decisions as to who accesses these are crucial.

In this article, we provide a rapid review of the key issues emerging in discussions about COVID and disability and discuss their relevance for triage and other procedures in South Africa. However, the issues arising in South Africa may be broadly relevant to other countries, especially low- to medium-income countries.

The UN’s *Disability-Inclusive Response to COVID-19*, published in May 2020, states that disabled people should be included in COVID-19 responses, which is in line with international commitments. These include the *United Nations Convention of the Rights of Persons with Disabilities* (UNCRPD), the *2030 Agenda for Sustainable Development* and the *Agenda for Humanity* (2016) and the *United Nations Disability Inclusion Strategy*. The UN’s stance emphasises that non-discrimination is a fundamental right for all people, and for this reason COVID-19 responses must ensure that they are not biased against disability (UN 2020). According to the UNCRPD, disabled people have equal rights to access to healthcare, and any denial of healthcare or health services on the basis of disability is discriminatory (Article 25 read with Article 2). It further highlights that disabled people should receive effective justice on an equal basis with others (Article 13). This raises the question as to why disabled people should not be regarded as equal in terms of access to ventilators and intensive care unit (ICU) admission. Decisions may be influenced by how society and policymakers regard disability, specifically the worth and value they attach to the lives of disabled people (Emanuel et al. 2020; Kittay 2020). Kittay (2020) shared her concerns:

Rationing and triage and isolation protocols aggravate my already stomach-churning fear. Even in the absence of overt discrimination, I and others like me must be concerned about the many ways discrimination is baked into standard practices and protocols. There are poison pills in seeming rational recommendations. (p. 1)

Ne’eman (2020:1) indicates that there is a real fear amongst disabled people that they will be overlooked, and suggests that they should object to having ‘second-class medical status’. However, there is both a local and a global disconnect between those who work on disability issues and are familiar with disability policies such as the UNCRPD and healthcare workers, who are often not trained in or familiar with these policies (Liasidou & Mavrou 2017). As a result, there was a lack of in-depth understanding of and training about disability and human rights even prior to the COVID-19 pandemic. This results in critical time opportunities being lost when rapid responses and intervention plans are being put in place (Qi & Hu 2020).

At the time of writing, South Africa is the epicentre of the COVID pandemic in Africa, with a disproportionately high number of cases in the Western Cape Province. In some African countries, very few people, if any at all, will gain access to life-saving care in the context of the epidemic. For example, the only African countries to have more than five ICU beds per 100 000 population are South Africa, Seychelles and Egypt (Ma & Vervoort 2020), with approximately 3450 ICU beds available in South Africa (population approximately 59 million) for COVID patients (Nichols et al. 2020). Complicating this, South Africa remains a deeply unequal society, as we discuss below, which may render triage considerations more complex; in South Africa there are resources but these are maldistributed, with far greater expenditure on healthcare provision in the small private healthcare sector than in the public sector, which caters to the bulk of the population (Harris et al. 2011; McIntyre 2019; Mcintyre & Klugman 2003; Mofolo, Heunis & Kigozi 2019). A recent research project has explored the question of how relatively greater prosperity in African countries may widen a number of access gaps between disabled and non-disabled people (Groce, Kett, Lang & Trani 2011); South Africa is an interesting case to consider because of its persistent and enduring high level of inequality. Before we turn specifically to the South African case, we review key issues about COVID and disability that are currently being discussed.

## Heightened risk

According to Pineda and Corburn (2020), disabled people living in cities during COVID-19 may be four times more likely to be injured or die than non-disabled people. They credit this not to disabled people’s inherent vulnerabilities, but rather to health policies, planning and practice that do not take the specific needs of disabled people into account. Disaggregated data by disability for COVID-related deaths are not, as far as we have been able to ascertain, currently available; a recent study conducted by the Office for National Statistics, United Kingdom, in England and Wales estimates the risk of death from COVID-19 for people with disabilities to be approximately double that of people without disabilities (https://www.ons.gov.uk/peoplepopulationandcommunity/birthsdeathsandmarriages/deaths/articles/coronaviruscovid19relateddeathsbydisabilitystatusenglandandwales/2marchto15may2020).

Disabled people are at an increased risk of contracting COVID-19 for a number of reasons, including difficulty with using basic protection measures and adhering to requirements set for social distancing. These difficulties include a lack of accessibility to water, sanitation and hygiene facilities. For example, the majority of disabled people live in homes without access to running water (Groce et al. 2011; Grut et al. 2012). Furthermore, many homes that do have running water have taps and basins that are inaccessible to the disabled people living there. For other disabled people, the act of handwashing as per COVID-19 guidelines is simply physically difficult or impossible.

Some disabled people require frequent physical contact with others to obtain the support they require (such as carrying, lifting or feeding by care assistants), which becomes challenging in the context of social distancing and self-isolation (Kuper et al. 2020; Mulibana 2020). Other disabled people are at a higher risk of contracting COVID-19 because of a lack of access to information regarding transmission and prevention of the virus, for example, healthcare information being broadcast in inaccessible formats, such as a lack of sign language interpreter, or the level of information being too complicated for someone with a learning disability to follow (Kuper et al. 2020; Mulibana 2020). Some disabled people are reliant on skin-to-surface touch for daily life, for instance, feeling the buttons on an elevator for someone with a visual impairment. Others, including those with psychosocial impairments, may reside in overcrowded or unsanitary institutional settings, which can increase their risk of infection.

In South Africa, during the initial stages of strict lockdown, vital disability-specific health services were not regarded as ‘essential services’, and this placed disabled people at heightened risk (Mulibana 2020). Health services such as sign language interpretation services for people who were deaf, assistive device and technology services, rehabilitation services, and therapeutic and developmental interventions were not regarded as essential (McKinney, McKinney & Swartz 2020; Mulibana 2020). The issue of South African Sign Language interpretation during COVID-19 has recently been raised:

The medical challenges deaf people experience are usually due to the fact that hospitals, doctors and nurses don’t know or understand sign language. The deaf patient therefore needs to rely on an interpreter which isn’t always possible due to availability and cost. (Huisman 2020:1)

In addition, some care homes and institutions for disabled people were closed, and disabled people were sent home to reside with their families, many of whom did not have the skills or knowledge of how to care for and stimulate their family members with disabilities (Mulibana 2020).

As mentioned earlier, there is a strong link between disability and poverty, which leads to the majority of disabled people residing in informal settlements in South Africa, where the risks of contracting COVID-19 are amplified (Armitage & Nellums 2020; Emmett 2006; Landes, Stevens & Turk 2020; UN 2020). Although disabled people are at heightened risk of dying if they contract COVID-19, they are also ‘in danger of being de-prioritised for care’ (Kuper et al. 2020:79).

On 26 March 2020, the World Health Organization (WHO 2020) developed a document, *Considerations for Disabled People during COVID-19*, that includes actions that need to be taken to ensure that disabled people are able to access healthcare services, water and sanitation services and public health information. However, the majority of these are not fully feasible in countries such as South Africa. For example, suggestions are made to make purchases online to buy essential items such as food and medicines (WHO 2020:3). This suggestion is not suitable for the majority of disabled people in South Africa, who do not have access to resources. The majority of disabled people cannot make online purchases as they have no credit cards or funds available, cannot access online shopping platforms because of a lack of Internet or devices, or reside in informal settlements where deliveries are not made (Emmett 2006; Groce et al. 2011). Disabled people are also encouraged to ensure that assistive devices, such as wheelchairs, crutches, walkers, transfer boards, white canes or other personal devices that are used on a daily basis, and especially in public spaces, are disinfected frequently (WHO 2020:3). However, this is also not possible for the majority of disabled South Africans, who continually struggle to find money for food and simply do not have the funds available, or the ability, to purchase expensive cleaning products (Mulibana 2020). In a recent interview, a woman wheelchair user who was the sole breadwinner of a household of six stated:

Most people buy one bottle of hand sanitiser, that will last them so long. We have to buy twice as much to sanitize my chair, too. It is so much responsibility. (Huisman 2020:1)

Additional challenges, besides regular safety and social distancing concerns, include not being able to buy essential products because of inaccessible public transport systems (Groce et al. 2011; Heap, Lorenzo & Thomas 2009). Disabled children are encouraged to continue playing, reading, learning and connecting with friends using telephone calls, texts or social media (WHO 2020:4). However, such activities may be extremely challenging when households have numerous family members all sharing a one-roomed dwelling with no food or electricity, let alone books or data to connect with friends (Emmett 2006; Grut et al. 2012).

## Persons with disabilities living in institutions are more likely to contract the virus and have higher rates of mortality

Disabled people, especially people with psychosocial and learning impairments, are at an increased risk of contracting COVID-19 as they are more likely than any other population group of comparable age to be institutionalised in nursing homes, psychiatric facilities, group homes, social care centres and even within prison facilities (Landes et al. 2020; UN 2020). At such institutions, there is often a heightened risk of spread of diseases and viruses because of challenges relating to implementing basic hygiene routines and maintaining social distancing, as well as limited access to accessible healthcare information, testing and appropriate healthcare provision (Armitage & Nellums 2020; Landes et al. 2020; Mulibana 2020; UN 2020). According to recent statistics, people residing in institutions are experiencing high numbers of COVID-19 infection, complications such as pneumonia and death (Comas-Herrera et al. 2020; Landes et al. 2020; UN 2020). It is for these reasons that COVID-19 policy responses, including triage protocols, need to be inclusive of disabled people in their design as well as implementation. In South Africa, those disabled people residing in institutions still in operation during lockdown are isolated from their family. Relatives have been prevented from visiting their disabled family members to protect them from the spread of the virus and are only permitted to make contact via the telephone, which is not suitable for some disabled people (Mulibana 2020). In a recent interview, a representative of Autism South Africa stated:

I know a mom who has not seen her teenage son since the lockdown because the residential facility will not allow her to visit. She can only phone. This is frustrating because her teenage son does not have a full functional speech. This really shows the lack of understanding because how are you expected to have a conversation when your child does not understand social communication? (Mulibana 2020:1)

## Triage

During settings such as disasters, when resources are limited and medical intervention and care are significant, healthcare workers are required to make decisions as to who can and who cannot access life-saving medical treatment. The prioritisation decisions are known as ‘triage’ and are most commonly used in emergency medicine situations, where there are many patients and few resources. During disasters, it is important that triage procedures be carefully decided upon to guide healthcare workers and standardise care (Sztajnkrycer, Madsen & Báez 2006; White & Lo 2020). Triage is a necessary process where need outstrips demand, and it is essential that triage decisions be based on the best available evidence (Auriemma et al. 2020; Joebges & Biller-Andorno 2020).

Researchers and ethicists have learned from disasters such as Hurricane Katrina in 2005 and the Haiti earthquake in 2010 and ascertained that there is an urgent need to establish clear triage policies that are standardised and assist healthcare workers in making life-or-death decisions (Klein et al. 2008; Sztajnkrycer et al. 2006). These triage protocols need to balance a number of competing considerations: healthcare workforce issues, duty to care, equal distribution amongst a population with diverse health needs, accountability of public departments and healthcare systems to serve the public interest, and preserving healthcare systems so that, after a disaster, recovery remains possible (Klein et al. 2008; Savin & Guidry-Grimes 2020). However, the implications of rationing life-saving resources during COVID-19 result in a situation where ‘the principle of “equals should be treated equally” may no longer be applicable’ (Mannelli (2020:364). In other words, choices will have to be made amongst people who are notionally equal, with some gaining access and others not.

While there exists a consensus that factors including a person’s gender, race and wealth should not play a role in determining inclusion criteria for accessing life-saving medical equipment and interventions, there remains a debate about whether disability should or should not be a consideration factor (Armitage & Nellums 2020; Emanuel et al. 2020).

Disabled people and their families are concerned that triage policies may devalue disabled people and exacerbate entrenched ableism within healthcare policy and practice. This, in turn, may lead to structural discrimination in the form of policies that directly or indirectly discriminate against disabled people (Kittay 2020; McKinney et al. 2020; Savin & Guidry-Grime 2020). Amongst the difficult triage decisions to be made in any scarce-resource context are questions about who is most likely to benefit from interventions that are not widely available. From a public health perspective, it makes no sense to offer expensive and scarce resources to those unlikely to benefit from them, and it is indeed the case that some disabled people, by reason of impairments and health conditions, may fall into this category, as would be the case for some non-disabled people. It is another matter, however, to assume that simply because a person has an impairment, it is automatically the case that that person would be less likely than others to benefit from scarce health resources. Triage should ideally operate as far as possible on the basis of evidence, rather than on the basis of assumptions about who can benefit. In writing about healthcare access in general for people with disabilities, it has been noted that it is important to avoid what has been termed ‘diagnostic over-shadowing’ (Shakespeare, Bright & Kuper 2018; Solomon et al. 2016). This refers to an assumption on the part of healthcare providers when treating disabled people that all health conditions experienced by them should be attributable to their impairments. By analogy, to make explicit or implicit triage decisions on the basis of disability status rather than on the basis of potential to benefit from treatment is a different, and problematic, form of over-shadowing.

## Triage types

It is important that triage policies be developed to provide clarity, consistency and fairness to decision-making relating to COVID-19 (Huxtable 2020). Regarding triage types, there are a number of triage guidelines, which are broadly based on four main models, namely, utilitarian (doing the greatest good for the greatest number of people), egalitarian (allocation based upon need), libertarian (protection of individual liberty and patient choice, including social benefit) and communitarian (respect for social and cultural values); the aspect of life cycle (fair innings or years life saved) is also considered (Armitage & Nellums 2020; Emanuel et al. 2020; Savin & Guidry-Grimes 2020).

The most current COVID-19 triage policies as used in a range of countries focus on the utilitarian view of saving more lives and more years of life (Emanuel et al. 2020; Savin & Guidry-Grimes 2020). Although the utilitarian view concentrates on societal good, it may place a burden of unacceptable sacrifice on individuals or groups of people, such as disabled people (White & Lo 2020). This triage framework deals with a key question: ‘Whose lives matter?’ Here, people with underlying comorbid conditions are excluded, as they may require more healthcare intervention and resources than those without. White and Lo (2020) suggest that these frameworks are ethically flawed, as the exclusion criteria used are selectively applied only to a specific group of people, rather than to all people who need critical medical care. In addition, this approach violates the principle of justice, as it applies different allocation criteria to separate groups of people and does not make clear what is ethically different from one group to another (Armitage & Nellums 2020; Savin & Guidry-Grimes 2020). As Kittay (2020:1) puts it, ‘benefits are not free-floating goods to be readily counted. Benefits attach to people’. A recently published paper noted that more lives may be saved if medical health professionals are permitted to exclude people who require more resources. However, no matter what triage type is used, some people will be excluded from receiving life-saving resources, which will result in them not surviving (Qi & Hu 2020; Mannelli 2020).

## Value and worth

When it comes to value and worth as a basis of triage, careful examination needs to be made as to whether the concepts of value and worth, however well-intentioned, may discriminate against disabled people (Armitage & Nellums 2020; Emanuel et al. 2020; Huxtable 2020). For decades, disabled people have been viewed as being inferior and their lives seen as less valuable than those of non-disabled people. Disabled people have been pitied, shamed and discriminated against on the basis of their disabilities (Savin & Guidry-Grimes 2020). Negative views towards disability have led to injustices in many forms, such as exclusion from education, employment and access to healthcare (McKinney, Lourens & Swartz 2018; Shakespeare 2017). When it comes to categorising and excluding groups of people, this may lead to some decision-makers feeling that the lives of disabled people have less worth than others and that their lives are ‘not worth saving’ (White & Lo 2020:1773). Eugenic views towards disability state that the world would be a better place if disability could be eliminated, whereas in direct contrast those holding a bioethical view see disability as being inherent in the human condition (Garland-Thomson 2012, 2017; Shakespeare 2017). Garland-Thomson states that disability affects all and ‘reflects the truth that we will all become disabled if we live long enough and that every life, every family has disability in it at some time’ (2012:339). From a bioethical view, disability is a natural part of humanity and of diversity. Eva Feder Kittay (2020), a professor emerita of philosophy at Stony Brook University and the mother of a daughter with a significant cognitive disability, noted in a recent article that, although her doctors said that her daughter has ‘no measurable IQ’:

[*S*]he lights up my life and the lives of those who get to know her. She loves her life, which is filled with music and joy. Her calm, steady loveliness makes the world a more beautiful place. (p. 1)

## The current South African context of care

On 15 March 2020, a national lockdown was declared in South Africa. Since then, COVID-19 positive cases have continued to rise on a daily basis.

As of 10 July 2020, 238 339 positive cases of COVID-19 have been identified, with 3720 deaths having been reported. The number of COVID-19 recoveries is currently 113 061, translating to a recovery rate of 47.4%; however, South Africa is moving into midwinter and the number of infections is forecasted to increase significantly (National Institute for Communicable Diseases [NICD] 2020b). Of these statistics, 31.4% of South Africa’s positive cases, and 2229 of the 3720 deaths, have been located within the Western Cape Province of South Africa, where the triage policy tool that will be later discussed has been adopted (NICD 2020b).

There is a significant risk that as the number of cases rise, the healthcare system could be overwhelmed (NICD 2020a). This will result in urgent critical care triaging decisions having to be made in both the government and private healthcare sectors. Although these decisions are crucial, they also raise significant ethical issues around who is able to, and who should be able to, access care (Kittay 2020; Kuper et al. 2020; Singh & Moodley 2020). Regarding policy responses to the COVID-19 pandemic in South Africa, there are numerous considerations that need to be taken into account. Many of these stem from the inequalities that were created during the apartheid regime, especially socio-economic disparities that are still felt today. For example, it is estimated that 55% of South Africans, or 30.4 million, live in poverty. As we have noted, there is a strong link between disability and poverty (Eide & Ingstad 2013; Groce et al. 2011; Statistics South Africa 2017). Moreover, research indicates that in addition to prevalent prejudice related to race, gender and socio-economic factors, disabled people experience discrimination based on their disabilities. This includes a lack of access to education or appropriate support within schools (Fleisch, Shindler & Perry 2012), lack of access to employment opportunities (McKinney & Swartz 2020) and a lack of access to healthcare (Maart & Jelsma 2014; Mji et al. 2017).

As a result of the multiple levels of inequality, COVID-19 responses are likely to have an unequal impact within differing contexts and amongst a diverse range of South Africans, and these issues need to be consciously addressed (Law Trust Chair in Social Justice 2020). The majority of disabled people live from hand to mouth and often rely on other people for care as well as limited social grants. Throughout the COVID-19 epidemic in South Africa, critical questions will be raised regarding what criteria should be used to guide rationing decisions when the demand for ventilators and ICU beds far exceeds the supply. Existing critical care resource recommendations, though carefully thought out, may remain ethically problematic as they involve prioritising certain groups of people over others. It is important that such factors be considered during the designing of triage policies (Sztajnkrycer et al. 2006; White & Lo 2020). In countries where all people have equal access to transport, first-come, first-served policy is seen as a ‘fairer’ system of triage. However, in a country like South Africa, this would not be ‘fair’ for most South Africans, especially those who depend on an unreliable public transportation system. Furthermore, this model would be even more discriminatory against disabled people, who cannot access most public transport systems or move freely within the South African built environment. Moreover, with the majority of disabled people being unemployed, they would not be able to afford to have their own private vehicles, hire transport from friends, family or community members or even pay for (unreliable) public transport. For example, minibus taxis are the most popular and common mode of transportation in South Africa. However, most minibus taxi operators will not stop along their busy routes to collect wheelchair users, let alone assist them to board and disembark the minibus taxi. If and when they do let them on board, operators are prone to charge wheelchair users a double fee, which they ‘justify’ because a wheelchair occupies the space of an additional paying passenger (Heap et al. 2009; Sherry 2015). In a recently published article, a reporter interviewed a South African wheelchair user about her experiences of using minibus taxi transportation during the pandemic: ‘Fellow passengers are loath to help her for fear of contracting the virus by touching her wheelchair’ (Huisman 2020:1). In addition, the national rail service, which represents the other preferred form of commuting, has been suspended because of the lockdown, so at the time of writing nobody is able to travel by train.

Although specific COVID-19 policy responses within South Africa have been developed to guide healthcare workers, including the *National Infection Prevention and Control Strategic Framework* (Department of Health [DoH] 2020), *Allocation of Scarce Critical Care Resources during the COVID-19 Public Health Emergency in South Africa* (Critical Care Society South Africa [CCSSA] 2020a) and the *Coronavirus Disease 2019 (COVID-19) Quick Reference for Clinical Health Care Workers* (National Institute for Communicable Diseases [NICS] 2020a), none of these documents speaks directly to disability.

## South African triage tools

To prioritise access to ICU facilities and ventilator support, the Western Cape government published the *COVID Critical Care Triage and Decision Tool* (Western Cape Government 2020a) and the *COVID-19 Outbreak Response Guidelines* in April 2020 (Western Cape Government 2020b). These documents provide healthcare workers with helpful standardised guidelines on the approach to managing the outbreak of COVID-19 in the Western Cape. We unfortunately do not have information on the extent to which these guidelines are followed in practice, and practices may change even within the same facility, but the way in which the guidelines are framed is instructive. However, our concern is shared by the South African Disability Alliance (SADA), and an investigative report has recently been submitted to the Ministry of Health (SADA 2020), raising urgent concerns that disabled people will not receive equal access to care. The guidelines express three main objectives: maintaining a standard of quality critical care, directing scarce critical care resources as efficiently and efficaciously as possible and providing a coordinated and consistent approach for public hospitals across the Western Cape. The document states that it conforms to the ethical duties of non-maleficence (duty to do no harm and to prevent harm), distributive justice (fair distribution of benefits and burdens) and autonomy (the ability to make one’s own decisions). The triage policy *Allocation of Scarce Critical Care Resources during the COVID-19 Public Health Emergency in South Africa* is based on the principles of ‘saving the most lives’ and ‘saving the most life years’. It uses the Clinical Frailty Scale, which includes a scale from 1 to 9, with 1 being those who are very fit and 9 including people who are terminally ill (from other causes) and approaching the end of life (CCSSA 2020a). However, disabled people may not be given priority or access to ICU care or ventilators because of the triage criteria discussed below.

Certain disabled people may be classified under category 4, ‘vulnerable’,which includes people who are not dependent on others for daily assistance but often have symptoms that limit activities, such as being ‘slowed up’ or being tired during the day. Those who fall under category 5, ‘mildly frail’ (in the CCSSA document there is an image of a person using a walker), are described as being those who require help with higher order instrumental activities of daily living (IADL), including finances, transportation, heavy housework, medications that would impact their ability to shop and walk outdoors independently, as well as preparation of food. People who are ‘classified’ as falling into category 6, ‘moderately frail’, include those who ‘need help with all outside activities and with keeping house’. This will include many people with disabilities who may have ‘problems with stairs, require assistance when bathing and may need minimal assistance (cuing, standby) with dressing’ (CCSSA 2020a:2). According to the SADA, the criteria of the triage document are claimed to be based on the prognosis of a patient. However, they believe that the issue of prognosis (and the implicit key question of whether a patient is likely to benefit from care interventions) is not sufficiently addressed and remains ‘completely subjective, without any regard to an evidence-based decision making process’ (SADA 2020:3). They further state that the use of the Clinical Frailty Scale does not take into account people with disabilities who may have a life expectancy equal to that of an able-bodied person, be very fit and yet be classified as severely frail because of a physical impairment. In addition, they state that this would be the same for a patient with intellectual disability, who may require full-time care and who would also be classified as severely frail (SADA 2020).

While disabled people scoring less than six will not be immediately excluded, they are still required to be prioritised via a second triage system based on the Sequential Organ Failure Assessment (SOFA) scale, which is based on the prognosis for short-term survival, as well as the comorbidity scores for long-term survival prognosis (CCSSA 2020a:1). Combined, these scores prioritise people as ‘red’ (scores of 1 to 3); ‘orange’ (scores of 4 and 5) and ‘yellow’ (scores of 6 to 8). If one follows the triage protocol, people classified as red would receive priority in accessing ventilator support, while a person with a priority score of yellow would have the lowest priority in accessing a ventilator and would receive resources only if they were still available after all patients classified as red and orange had been accommodated (CCSSA 2020a). If there are ties within the same colour grouping, then priority would be given to those youngest in age or individuals whose work supports the provision of acute care to others, together with lower priority scores.

Although there is no specific mention of disability, category 7, ‘severely frail’, describes those who are completely dependent for personal care, from whatever cause (physical or cognitive). Even so, people who seem stable and not at high risk of dying (within 6 months) are included in this category. Also included in this description is a silhouetted image of a person being pushed in a wheelchair, a symbol strongly associated with disability worldwide, to illustrate what category of person would be included in this group (by contrast, under Category 1, ‘very fit’, the associated image is an upright silhouette of a person running). These images and the accompanying descriptions raise the question as to how people who are wheelchair users are perceived, especially within emergency healthcare situations. Furthermore, which assumptions do they express about those disabled people who are completely reliant on personal care but who are healthy and are not expected to die within 6 months (McKinney et al. 2020)?

As mentioned above, people with a Frailty Assessment Score of less than 6 will not receive ventilators or be able to access the ICU. They will instead receive a management plan, which includes isolation in a COVID-19 isolation ward and discussions of end-of-life issues with next of kin. If a person’s health deteriorates or no improvements are seen, the triage plan moves on to ‘end-of-life care where palliative care teams will provide additional support and consultation’ (CCSSA 2020a:1).

Since earlier versions of this article were written, the CCSSA guidelines have now been updated. There is now a note added, which reads ‘[*t*]he Clinical Frailty Scale (CFS) [*sic*] is not applicable in patients with stable long-term disabilities (for example, cerebral palsy), learning disabilities or autism’ (CCSSA 2020b:2). This is a very welcome addition and an important one. The fact that it is a late edition, though, does show the conflation of disability and frailty in the original version, and the changes may not be fully clear to all using the guidelines. It also uses the term ‘learning disabilities’, which in South Africa is often used as a term distinct from ‘intellectual disability’, unlike in Britain, where the terms are synonymous. South African users of the guidelines may still regard people with intellectual disabilities as covered by the CFS.^1^

## Development of triage policies and involvement of stakeholders

It is important that COVID-19 policies and responses, and the implementation of these, be monitored to ensure that they are inclusive of all people, especially those from vulnerable groups, including disabled people. To do this, policies and responses need to be developed with ethical and legal input via a collaborative team of experts as well as stakeholders from government, academia and civil society (Huxtable 2020). These need to be ‘multidimensional, multifactorial matrix decision-making processes’ that can be used by healthcare workers (Klein et al. 2008:2). In addition, two-way communication between government and society is highlighted as being essential during COVID-19 to ensure accountability as well as public buy-in and trust, which is formed via inclusive participation. Citizens need to feel that their concerns have been raised and that their voices are heard (Huxtable 2020; Kuper et al. 2020; Law Trust Chair in Social Justice 2020).

In South Africa, a working group consisting of social justice practitioners and activists from civil society and the academic community, such as the Law Trust Chair in Social Justice, Stellenbosch University, has been established and aims to assist the government in monitoring the implementation of the COVID-19 policies. This working group focuses on identifying, reviewing and assessing COVID-19 policies and responses to these policies, ensuring that they reflect equal enjoyment of all rights and freedoms, as well as the rule of law and peace for all people. The group aims to ensure responsiveness to the lived experiences of the most vulnerable communities across South Africa to make certain that implementation does not undermine the achievement of equality, human dignity and advancement of human rights and freedoms for all, which also assists in creating social accountability (Law Trust Chair in Social Justice 2020).

Stakeholder participation, specifically from disability organisations, has thus far taken the following form, to the best of our knowledge. The Presidential Working Group on Disability, which is an existing advisory body to the president regarding the implementation of disability policy, together with two disabled people’s organisations, SADA and Disabled People South Africa (DPSA), joined a webinar hosted by the Ministry of Women, Youth, and Persons with Disabilities on 22 May 2020 regarding the implementation of policy relating to COVID-19 and disabled people. In addition, disabled people have been represented in the government’s COVID-19 Crisis Committee. However, although interaction between these organisations and the state is taking place, questions as to what impact has been made in the lives of disabled people, and whether their needs have been included, have been raised (Blind SA 2020; Mulibana 2020; SADA 2020). It has been stated that although COVID-19 disaster management committees were established prior to lockdown, no inclusion of disability rights coordinating mechanisms took place and that overall, disability issues have been neglected in COVID-19 disaster management responses (Mulibana 2020; SADA 2020). During a media briefing responding to the webinar hosted by the Ministry of Women, Youth and Persons with Disability, an umbrella organisation for people with visual impairments, Blind SA, stated that many of the commitments made by South Africa’s president, as well as the ministry, have not been realised and that during COVID-19 these will be given even lower priority. Blind SA (2020) further stated that they were disappointed at the ministry’s response, stating that there were incomplete proposals from government in terms of ensuring disability mainstreaming and support. They wanted these addressed as a matter of urgency.

When it comes to the application and interpretation of *Western Cape Provincial Critical Care Decision Tool*, the Western Cape Network on Disability recently submitted an enquiry to the SADA. A SADA task team was established to review the tool, and the following issues were raised for investigation: the interpretation of the tool, the wider applicability of the tool to other provinces in South Africa and the availability of international instruments offering guidance in critical care decision-making processes. The investigatory report made a number of recommendations to the Minister for Health, including the need for mainstreaming disability in respect of all COVID-19 responsive programmes; for information to be made available and facilities, services and programmes made accessible to all disabled people; for constant engagement with the organisations representing disabled people to ensure meaningful participation throughout the processes of COVID-19 recovery; for government and the sector to develop an accountability mechanism to ensure monitoring of progress in response to COVID-19 recovery plans; for institutionalised patients to be provided with maximum support through institution-specific programmes, including preventive measures and testing; and finally for a meeting with the minister to be held (SADA 2020). However, currently no feedback has been given from the ministry.

## Conclusion

While some countries may use triage criteria that are based on the perceived worth of a person’s life and their ability to contribute to society, which may discriminate against disabled people if they are viewed from a medical perspective of being ‘less able’, some current South African triage policies, in our reading, completely exclude many disabled people, especially those with physical disabilities. We understand that triage is always difficult and that the reality is that in South Africa many non-disabled people will also not gain access to care. However, this overall contextual reality does not make irrelevant the broader question of discrimination against disabled people, which has always, to varying degrees, been a life-and-death issue but is now much more acutely so.

The *Bill of Rights*, which forms part of the *Constitution of the Republic of South Africa* (1996), states that all people, including disabled people, are equal, that everyone has the right to access healthcare services, that everyone has inherent dignity and the right to have their dignity respected and protected, and that everyone has the right to life. Although all individual rights are subject to limitations under certain circumstances,^2^ if many disabled people are excluded from receiving life-saving support during COVID-19, what will South Africa look like after the pandemic? As Ne’eman (2020:1) states, ‘[*t*]he ranks of the survivors would look very different, biased toward those who lacked disabilities before the pandemic. Equity would have been sacrificed in the name of efficiency’.

We cannot and do not pretend to have all the answers for difficult triage decisions, some of which are likely to be made informally and on the spur of the moment. Nonetheless, we do believe that at this time, it is important that people be aware of the issues at stake.

## Footnotes

1 In our reading of the revised CCSSA guidelines (Version 3), it appears that the change to the guidelines may have been made in response to changes in the British NICE guidelines *COVID-19 Rapid Guideline: Critical Care in Adults* (NICE guideline [NG159]; https://www.nice.org.uk/guidance/NG159). This guideline has in its Critical Care Admission Algorithm (https://www.nice.org.uk/guidance/ng159/resources/critical-care-admission-algorithm-pdf-8708948893) the following text: ‘Any patient aged under 65, or patient of any age with stable long-term disabilities (for example, cerebral palsy), learning disabilities or autism: do an individualised assessment of frailty. Do not use CFS score’. In our reading of Version 3 of the CCSSA guidelines, the NICE recommendation that an individualised assessment of frailty be undertaken is not reproduced. This may possibly also lead to some confusion.

2 In the case of *Soobramoney versus the Minister of Health* (KZN), Case no. CCT32/97, the Constitutional Court asserted that in the case of use of scarce resources, the state must apply ‘apply a holistic approach to the larger needs of society rather than to focus on specific needs of particular individuals within society’ (Paragraph 31).
